# Genome-wide identification and expression analysis of wall-associated kinase (WAK) and WAK-like kinase gene family in response to tomato yellow leaf curl virus infection in *Nicotiana benthamiana*

**DOI:** 10.1186/s12870-023-04112-2

**Published:** 2023-03-17

**Authors:** Xueting Zhong, Jiapeng Li, Lianlian Yang, Xiaoyin Wu, Hong Xu, Tao Hu, Yajun Wang, Yaqin Wang, Zhanqi Wang

**Affiliations:** 1 Key Laboratory of Vector Biology and Pathogen Control of Zhejiang Province, College of Life Sciences, Huzhou University, Huzhou, 313000 China; 2 State Key Laboratory of Rice Biology, Institute of Biotechnology, Zhejiang University, Hangzhou, 310058 China

**Keywords:** *Nicotiana benthamiana*, *WAK/WAKL* gene family, Phylogenetics, TYLCV infection, Expression profile, Stress response

## Abstract

**Background:**

Tomato yellow leaf curl virus (TYLCV) is a major monopartite virus in the family *Geminiviridae* and has caused severe yield losses in tomato and tobacco planting areas worldwide. Wall-associated kinases (WAKs) and WAK-like kinases (WAKLs) are a subfamily of the receptor-like kinase family implicated in cell wall signaling and transmitting extracellular signals to the cytoplasm, thereby regulating plant growth and development and resistance to abiotic and biotic stresses. Recently, many studies on *WAK/WAKL* family genes have been performed in various plants under different stresses; however, identification and functional survey of the *WAK/WAKL* gene family of *Nicotiana benthamiana* have not yet been performed, even though its genome has been sequenced for several years. Therefore, in this study, we aimed to identify the *WAK/WAKL* gene family in *N. benthamiana* and explore their possible functions in response to TYLCV infection.

**Results:**

Thirty-eight putative *WAK/WAKL* genes were identified and named according to their locations in *N. benthamiana*. Phylogenetic analysis showed that *NbWAK/WAKLs* are clustered into five groups. The protein motifs and gene structure compositions of *NbWAK/WAKLs* appear to be highly conserved among the phylogenetic groups. Numerous cis-acting elements involved in phytohormone and/or stress responses were detected in the promoter regions of *NbWAK/WAKLs*. Moreover, gene expression analysis revealed that most of the *NbWAK/WAKLs* are expressed in at least one of the examined tissues, suggesting their possible roles in regulating the growth and development of plants. Virus-induced gene silencing and quantitative PCR analyses demonstrated that *NbWAK/WAKLs* are implicated in regulating the response of *N. benthamiana* to TYLCV, ten of which were dramatically upregulated in locally or systemically infected leaves of *N. benthamiana* following TYLCV infection.

**Conclusions:**

Our study lays an essential base for the further exploration of the potential functions of *NbWAK/WAKLs* in plant growth and development and response to viral infections in *N. benthamiana*.

**Supplementary Information:**

The online version contains supplementary material available at 10.1186/s12870-023-04112-2.

## Background

The *Geminiviridae* are a large family of plant viruses with circular single-stranded DNA genomes that can infect cash and food crops, resulting in substantial economic losses worldwide [1, 2]. Geminiviruses are currently classified into 14 genera according to their host range, transmission vector, and genome organization, including *Becurtovirus*, *Begomovirus*, *Capulavirus*, *Citlodavirus*, *Curtovirus*, *Eragrovirus*, *Grablovirus*, *Maldovirus*, *Mastrevirus*, *Mulcrilevirus*, *Opunvirus*, *Topilevirus*, *Topocuvirus*, and *Turncurtovirus* [3]. Plants infected with geminiviruses frequently exhibit various symptoms, including leaf curl, chlorosis, shoot twisting, stunting, fruit distortion, and plant death, ultimately leading to huge yield losses [4, 5]. Over the past few decades, geminiviruses have spread rapidly around the world due to the high rates of replication, mutation, and recombination in their genomes [6–8]. Every year, the economic losses caused by emerging geminiviruses are estimated to be several hundred million dollars, especially in Africa and Asia [4, 9]. Therefore, geminiviruses have been recognized as a serious threat to global agriculture and food security.

Tomato yellow leaf curl virus (TYLCV), a monopartite begomovirus in the family *Geminiviridae*, has spread worldwide [10]. This virus is a major global virus that causes a severe yellow leaf curl disease and is responsible for significant yield losses in tomato and tobacco planting areas [10, 11]. Recent studies showed that the genome of TYLCV contains eight open reading frames, namely, V1, V2, V3, C1, C2, C3, C4, and C5 [12–14]. TYLCV V3 is an endoplasmic reticulum- and Golgi-localized protein that suppresses host RNA silencing and trafficking of virions between cells in host plants [12, 13]. TYLCV AC5 is a symptom determinant and viral suppressor of RNA silencing, which can inhibit transcriptional gene silencing (TGS) and post-transcriptional gene silencing (PTGS) and increase the pathogenicity of TYLCV to enhance the success of viral infection [14]. TYLCV V2 is a multifunctional protein that has been shown to interact with the host suppressor of gene silencing 3 to inhibit the PTGS [15] and with histone deacetylase 6 and argonaute 4 to inhibit methylation-mediated TGS in plants [16, 17]. Recently, TYLCV V2 was also reported to be involved in the nuclear export of V1, which is critical for the viral spread and systemic infection of TYLCV [18]. Finally, TYLCV C4 is a double-localized protein that interacts with a broad range of plant receptor-like kinases (RLKs) to prevent the cell-to-cell spread of RNA silencing [19, 20]. Additionally, overexpression of TYLCV C4 in *Arabidopsis* confers drought tolerance via an abscisic acid (ABA)-independent mechanism [21], and transgenic expression of TYLCV C4 in tomatoes leads to an alteration in the expression of plant developmental genes responsible for leaf upward cupping phenotype [22]. Therefore, it seems that each of the TYLCV-encoded proteins plays a critical role in its pathogenicity, and much research is still needed to understand the pathogenic mechanism of TYLCV.

When plants face abiotic and biotic stress conditions, they can sense and transmit external stimulus signals intracellularly through a wide range of receptors and produce various adaptive responses to environmental stimuli [23, 24]. RLKs are a large family that transmits signals from the outside to the inside of the cell via their intracellular kinase domains [25, 26]. Wall-associated kinases (WAKs) are a subfamily of the RLK family that contains a transmembrane proprotein, a cytoplasmic serine/threonine kinase domain, and an epidermal growth factor (EGF)-like structural domain [26, 27]. Furthermore, WAK-like kinases (WAKLs) are a type of RLKs with a structure similar to that of WAKs in plants [28, 29]. WAKs and WAKLs (WAK/WAKLs) form an essential class of RLKs implicated in cell wall signal sensing and transmitting extracellular signals to the cytoplasm, thereby regulating plant growth and development and stress responses [30–32]. For example, in *Gossypium hirsutum*, *WAK/WAKL* genes are reported to be involved in cotton fiber growth by regulating auxin and gibberellin levels [33]. In *Arabidopsis*, AtWAKL10 negatively regulates leaf senescence, and overexpression of AtWAKL10 causes transcriptional alterations of a specific set of genes involved in cell extension and cell wall modification [34]. Furthermore, in *Arabidopsis* and rice beans, *WAK1s* are implicated in response to aluminum toxicity, and overexpression of *AtWAK1* can enhance plant tolerance to aluminum stress [35, 36]. In rice, *OsWAK11* is characterized to detoxify excessive copper, and the knockdown of *OsWAK11* results in hypersensitivity to copper toxicity [37].

In addition to the involvement in plant growth and development and responses to abiotic stresses, *WAK/WAKL* genes also play critical roles in plant responses to pathogen attacks [38, 39]. Recently, *WAK/WAKL* genes have been identified to prevent fungal and bacterial diseases in several crop species. For example, *Xa4* encodes a WAK XA4 in rice and confers durable resistance to *Xanthomonas oryzae* by strengthening the cell wall [40]. In wheat, *Stb6* encodes a conserved WAKL protein that manages gene-for-gene resistance against *Zymoseptoria tritici* in a hypersensitive response-independent manner [41]. Furthermore, *TaWAK2* has been reported to prevent penetration and spread of *Fusarium graminearum* by suppressing the expression of *pectin methyl esterase 1* to produce a more rigid cell wall [42]. In maize, it has also been shown that *qHSR1* and *Htn1*, which encode two WAK/WAKL proteins, are implicated in plant resistance against fungal pathogens *Sporisorium reilianum* and *Exserohilum turcicum*, respectively [43, 44]. Additionally, it has been demonstrated that SlWAK1 interacts with Fls2/Fls3 to include the deposition of callose in tomatoes and minimizes the pathogen infection with *Pseudomonas syringae* [45]. More recently, a specific set of *GhWAKLs* has been reported to be induced by *Verticillium dahliae* infection, and the knockdown of *GhWAKL* expression suppresses jasmonic acid (JA)- and salicylic acid (SA)-mediated defense responses, impairing the resistance of cotton to *Verticillium dahliae* [46, 47]. These pleiotropic effects on pathogen infections frequently provide additional economic and agronomic benefits for crops that possess these *WAK/WAKL* genes. However, whether *WAK/WAKLs* are implicated in plant defense against viral infections remains unclear.

In this study, the *WAK/WAKL* gene family in *Nicotiana benthamiana* was identified at a genome-wide level using the bioinformatics method, and their potential roles in response to TYLCV infection were investigated using a virus-induced gene silencing (VIGS) approach. We identified 38 *WAK/WAKL* family members in the *N. benthamiana* genome and found that 15 *WAK/WAKLs* are differentially expressed following TYLCV infection. Further VIGS analysis showed that disrupting the expression of *WAK/WAKLs* in *N. benthamiana* increases host susceptibility to TYLCV. Our results provide new evidence that *N. benthamiana WAK/WAKLs* (*NbWAK/WAKLs*) contribute to plant resistance to TYLCV infection. The study lays an essential base for further research on the potential functions of *NbWAK/WAKLs* in plant growth and development and response to viral infections in *N. benthamiana*. In the present study, we aimed to provide a comprehensive view of the *NbWAK/WAKL* gene family in *N. benthamiana* and to identify members involved in response to TYLCV infection.

## Results

### Identification of the *WAK/WAKL* gene family in *N. benthamiana*

In this study, a hidden Markov model (HMM) was constructed using WAK/WAKL protein sequences from *Arabidopsis* and tomato (Additional file 1) to identify the *WAK/WAKL* gene family *N. benthamiana*. As a result, 15 putative *NbWAK* genes and 23 putative *NbWAKL* genes were identified in *N. benthamiana* and designated as *NbWAK1*–*NbWAK15* and *NbWAKL1*–*NbWAKL23*, respectively, according to their locations in the genome (Table 1). The genomic DNA of the identified *NbWAK/WAKLs* ranged from 1305 to 13,178 bp. The amino acid residue numbers of the putative NbWAK and NbWAKL proteins varied from 202 to 1159, and their isoelectric point (pI) and molecular weight (MW) ranged from 4.9–9.4 and 22.3–128.2 kDa, respectively (Table 1).

**Table 1:** Structural and biochemical features of the *WAK/WAKL* gene family in *N. benthamiana*

Name	Gene ID^**a**^	Gene position	Strand	Size							
				Genomic DNA (bp)	mRNA (bp)	CDS^**b**^ (bp)	5′-UTR^**c**^ (bp)	5′-UTR^**d**^ (bp)	Protein (aa)	pI^**e**^	MW^**f**^ (kDa)
*NbWAK1*	Niben101Scf00149g10001	Niben101Scf00149: 1014401..1025094	forward	10,694	2912	2811	101	0	936	8.0	103.7
*NbWAK2*	Niben101Scf00149g10003	Niben101Scf00149: 1039140..1052317	reverse	13,178	1707	1707	0	0	568	6.7	62.0
*NbWAK3*	Niben101Scf00530g11020	Niben101Scf00530: 1143477..1146128	reverse	2652	2075	1917	42	116	638	7.2	72.2
*NbWAK4*	Niben101Scf01237g11009	Niben101Scf01237: 1118347..1122027	forward	3681	2205	2205	0	0	734	6.0	81.6
*NbWAK5*	Niben101Scf02160g02006	Niben101Scf02160: 217154..220237	reverse	3084	2707	1905	291	511	634	6.4	71.4
*NbWAK6*	Niben101Scf02608g01007	Niben101Scf02608: 112748..119830	forward	7083	2385	2115	270	0	704	6.8	77.6
*NbWAK7*	Niben101Scf02608g01008	Niben101Scf02608: 148832..157171	forward	8340	3204	2115	270	819	704	6.6	77.6
*NbWAK8*	Niben101Scf03202g03004	Niben101Scf03202: 362629..366940	forward	4312	2247	2247	0	0	748	6.1	82.6
*NbWAK9*	Niben101Scf03202g04015	Niben101Scf03202: 391694..396324	forward	4631	2202	2202	0	0	733	7.7	79.9
*NbWAK10*	Niben101Scf03202g04017	Niben101Scf03202: 397065..401394	reverse	4330	2157	2157	0	0	718	6.4	79.7
*NbWAK11*	Niben101Scf03472g00005	Niben101Scf03472: 41760..50789	reverse	9030	1953	1953	0	0	650	8.5	72.9
*NbWAK12*	Niben101Scf06394g08019	Niben101Scf06394: 877593..890273	forward	12,681	3083	2868	215	0	955	6.0	105.4
*NbWAK13*	Niben101Scf10330g02004	Niben101Scf10330: 231600..234369	reverse	2770	2206	1893	107	206	630	5.7	68.3
*NbWAK14*	Niben101Scf13018g00012	Niben101Scf13018: 12405..25275	reverse	12,871	3699	3480	219	0	1159	5.9	128.2
*NbWAK15*	Niben101Scf21196g00008	Niben101Scf21196: 20786..24874	forward	4089	1854	1854	0	0	617	8.3	69.0
*NbWAKL1*	Niben101Scf00149g10020	Niben101Scf00149:1031476..1037918	forward	6443	3469	1203	88	2178	400	5.1	42.9
*NbWAKL2*	Niben101Scf00327g01037	Niben101Scf00327:124864..127845	reverse	2982	2291	1941	122	228	646	8.5	72.5
*NbWAKL3*	Niben101Scf00530g11014	Niben101Scf00530:1137409..1142320	reverse	4912	3686	1509	1733	444	502	5.9	57.0
*NbWAKL4*	Niben101Scf00700g06021	Niben101Scf00700:668335..673932	reverse	5598	2661	2661	0	0	886	6.2	98.8
*NbWAKL5*	Niben101Scf01521g06001	Niben101Scf01521:684767..686339	reverse	1573	1476	915	194	367	304	4.9	33.6
*NbWAKL6*	Niben101Scf02290g02006	Niben101Scf02290:197165..201565	forward	4401	2052	1908	133	11	635	8.4	71.1
*NbWAKL7*	Niben101Scf02381g08016	Niben101Scf02381:792605..794693	reverse	2089	1023	1023	0	0	340	6.1	38.1
*NbWAKL8*	Niben101Scf03202g03002	Niben101Scf03202:301027..307217	forward	6191	1011	1011	0	0	336	9.4	37.7
*NbWAKL9*	Niben101Scf03304g01026	Niben101Scf03304:167027..172041	reverse	5015	2064	2064	0	0	687	6.2	76.2
*NbWAKL10*	Niben101Scf03363g01019	Niben101Scf03363:210073..212041	reverse	1969	1807	1029	90	688	342	8.3	39.0
*NbWAKL11*	Niben101Scf03445g00009	Niben101Scf03445:15851..17440	forward	1590	1176	843	333	0	280	5.5	30.5
*NbWAKL12*	Niben101Scf03939g06023	Niben101Scf03939:737517..742076	forward	4560	1395	1128	0	267	375	6.1	41.5
*NbWAKL13*	Niben101Scf04445g01002	Niben101Scf04445:135918..147686	reverse	11,769	2886	2886	0	0	961	8.5	107.5
*NbWAKL14*	Niben101Scf05368g07009	Niben101Scf05368:738506..740861	reverse	2356	1865	1584	0	281	527	6.3	59.0
*NbWAKL15*	Niben101Scf06394g08020	Niben101Scf06394:862041..863966	forward	1926	789	789	0	0	262	5.1	27.8
*NbWAKL16*	Niben101Scf06909g04005	Niben101Scf06909:411031..419054	reverse	8024	1899	1899	0	0	632	5.1	71.1
*NbWAKL17*	Niben101Scf07969g00010	Niben101Scf07969:42176..44432	forward	2257	1066	888	178	0	295	5.3	32.5
*NbWAKL18*	Niben101Scf11389g01034	Niben101Scf11389:144908..147896	forward	2989	1529	951	197	381	316	4.9	34.9
*NbWAKL19*	Niben101Scf11416g00017	Niben101Scf11416:76861..79712	reverse	2852	1899	1899	0	0	632	5.9	70.2
*NbWAKL20*	Niben101Scf14950g00001	Niben101Scf14950:25106..26461	reverse	1356	1040	864	176	0	287	5.3	31.4
*NbWAKL21*	Niben101Scf20037g00022	Niben101Scf20037:58700..62033	reverse	3334	2613	2103	168	342	700	7.9	77.1
*NbWAKL22*	Niben101Scf21589g00004	Niben101Scf21589:55217..57416	reverse	2200	1074	858	216	0	285	6.1	31.1
*NbWAKL23*	Niben101Ctg15342g00002	Niben101Ctg15342:201..1505	forward	1305	862	609	253	0	202	5.3	22.3

^a^ Gene ID, the gene locus in the Sol Genomics Network (https://solgenomics.net/). ^b^ *CDS* coding sequence. ^c^ *5*′*-UTR* 5′-untranslated region. ^d^ *3*′*-UTR* 3′-untranslated region. ^e^ pI, isoelectric point. ^f^ MW, molecular weight

### Phylogenetic analysis of NbWAK/WAKL proteins

**Fig. 1:**
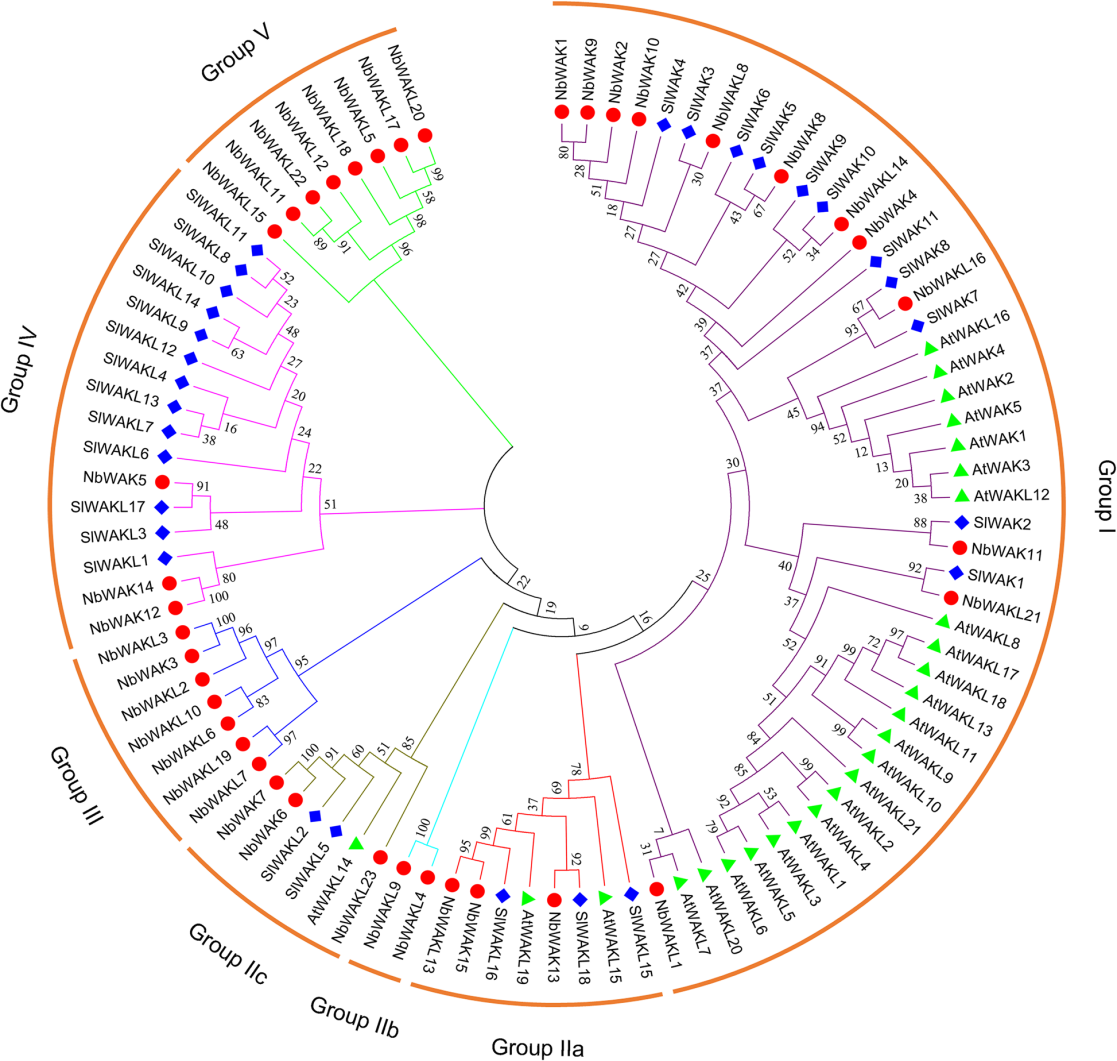
Phylogenetic analysis of wall-associated kinase (WAK) and WAK-like (WAKL) proteins from *Nicotiana benthamiana*, *Arabidopsis*, and tomato. A phylogenetic tree was constructed using the maximum-likelihood (ML) method with 1000 bootstrap replicates for each branch through MEGA 11.0. WAKs and WAKLs from different plant species are labeled with different colors. Purple, red, turquoise, green-brown, blue, pink, and green clusters represent Groups I, IIa, IIb, IIc, III, IV, and V, respectively

To determine the evolutionary relationships between the members in the *NbWAK/WAKL* gene family and further infer their putative functions based on homologous genes in other plants, we constructed a phylogenetic tree using 38 NbWAK/WAKLs together with 26 AtWAK/WAKLs [28] and 29 SlWAK/WAKLs [48]. As shown in Fig. 1, these 93 WAK/WAKL proteins were divided into five clusters (Groups I–V), and Group II was further classified into three subgroups (Groups IIa, IIb, and IIc). Group I consisted of 23 AtWAK/WAKL members, 12 NbWAK/WAKL members, and 11 SlWAK/WAKL members. Group II, the largest subgroup, comprised eight NbWAK/WAKL members, five SlWAK/WAKL members, and three AtWAK/WAKL members. Group III contained only seven members, NbWAK3 and NbWAKLs 2, 3, 6, 7, 10, and 19, and so was the smallest subgroup. Group IV possessed 13 NbWAK/WAKL and three SlWAK/WAKL members. Like Group III, Group V included eight members of NbWAK/WAKLs. Notably, Groups III and V were subgroups specific to *N. benthamiana* (Fig. 1). These results indicate that *N. benthamiana* has evolved many WAK/WAKLs during long-term acclimation.

### Conserved motif analysis of NbWAK/WAKL Proteins

**Fig. 2:**
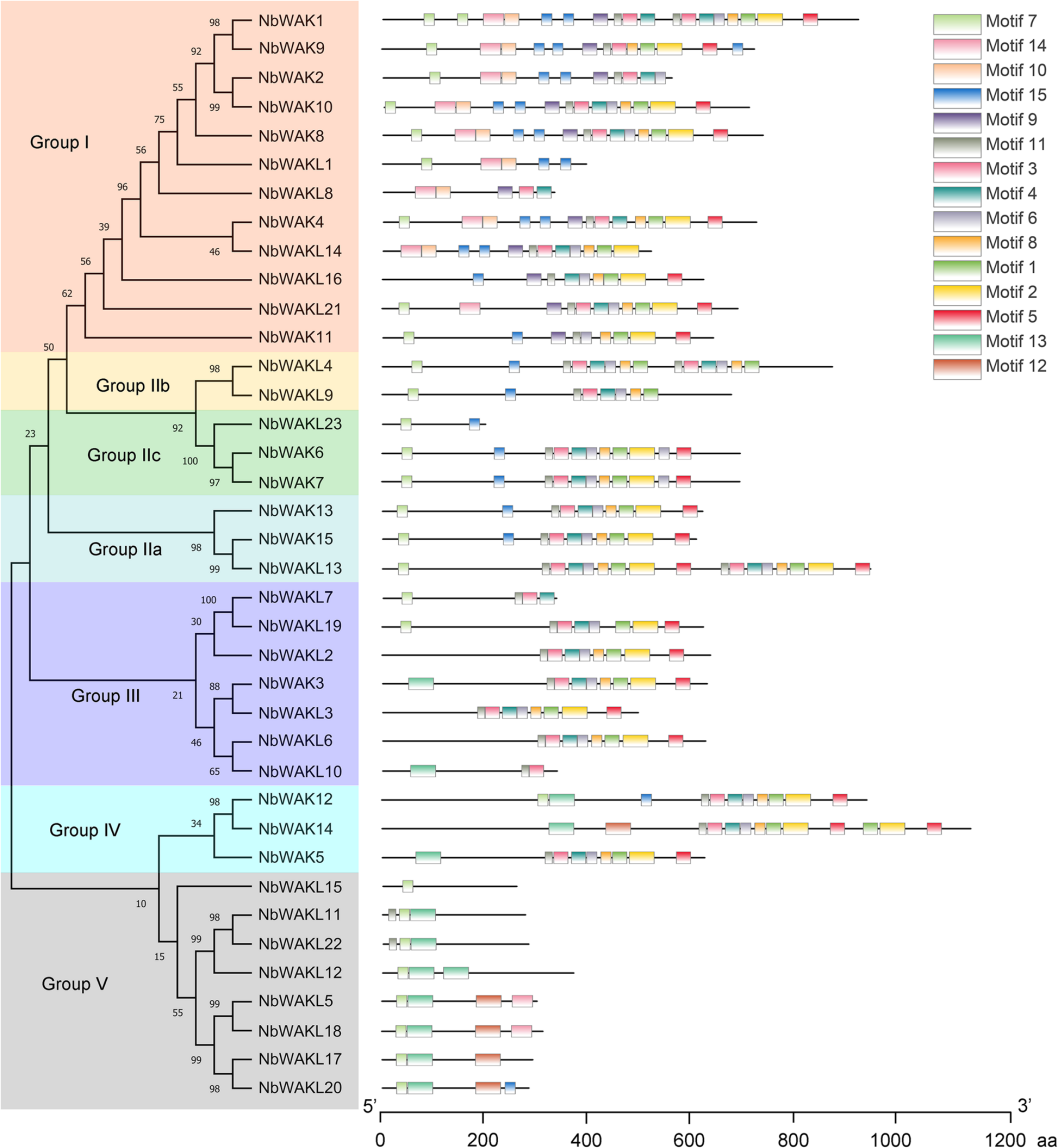
Schematic representation of molecular phylogenetic relationships and conserved motifs of WAK/WAKL proteins in *Nicotiana benthamiana*. The phylogenetic tree was constructed using the maximum-likelihood (ML) method with 1000 bootstrap replicates for each branch through MEGA 11.0. Conserved motifs were identified using the Multiple Em for Motif Elicitation (MEME) suite with the complete protein sequences of NbWAK/WAKLs and visualized using TBtools (v.1.098661). Different colored boxes indicate different motifs. Locations of different motifs are proportional to their sequence lengths

To fully understand the diverse functions of these NbWAK/WAKL proteins, their conserved motifs were analyzed using the Multiple Em for Motif Elicitation (MEME) suite (http://meme-suite.org/tools/meme/) [49]. As shown in Fig. 2, a total of 15 conserved motifs (Motifs 1–15) were detected in the protein sequences of NbWAK/WAKLs. MEME analysis and the phylogenetic tree indicated that motif structures of NbWAK/WAKLs varied considerably between members of different phylogenetic groups, but they were similar between members of the same group (Fig. 2). Furthermore, some motifs were unique to certain phylogenetic groups. For example, Motifs 9 and 10 were present only in Group I, and Motifs 12 and 13 were mainly detected in Group V (Fig. 2), suggesting that these specific motifs might contribute to the functional divergence of NbWAK/WAKLs in different groups. In addition, most of the structurally similar NbWAK/WAKLs were found to possess the common motifs (Fig. 2), indicating similar functions and functional divergence of these family members over their evolutionary courses.

### Gene structure analysis of *NbWAK/WAKL* genes

**Fig. 3:**
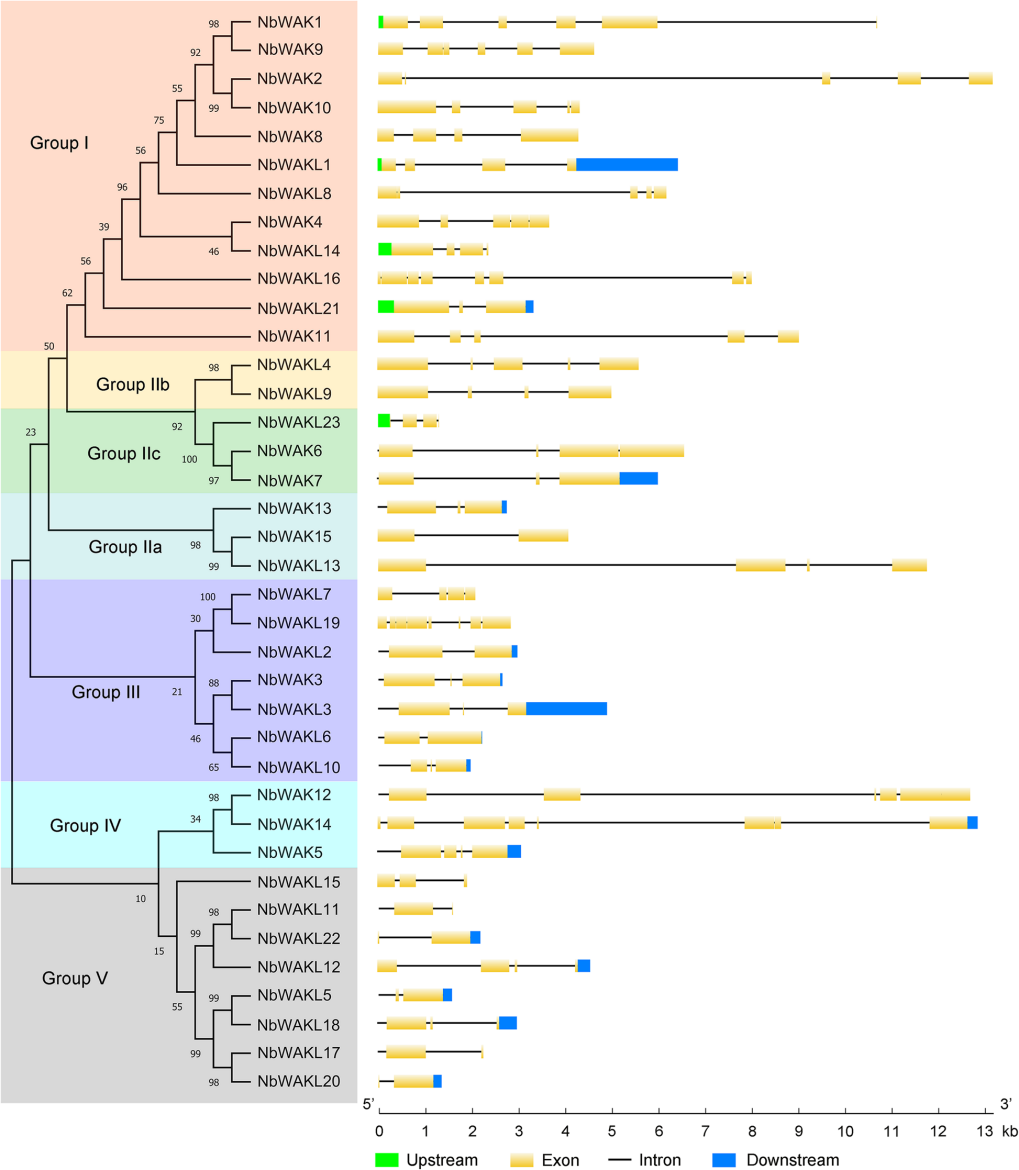
Schematic representation of molecular phylogenetic relationships and gene structure of *WAK/WAKLs* in *Nicotiana benthamiana*. The phylogenetic tree was constructed using the Maximum-likelihood method with 1000 bootstrap replicates for each branch through MEGA 11.0. The diagrammatic genomic organization of *NbWAK/WAKLs* was produced using the Gene Structure Display Server (GSDS) 2.0. Upstream sequences, exons, introns, and downstream sequences are indicated by green boxes, yellow boxes, black lines, and blue boxes, respectively

To obtain more information about the structural diversity of *NbWAK/WAKL* genes, we constructed a maximum-likelihood (ML) phylogenetic tree and analyzed the exon–intron organization of *NbWAK/WAKL* genes using the Gene Structure Display Server 2.0 (GSDS 2.0) (http://gsds.gao-lab.org/) web portal [50]. As shown in Fig. 3, *NbWAK/WAKLs* had 2–8 exons, with the most exons (eight exons) found in *NbWAK14*, *NbWAKL16*, and *NbWAKL19* and the least (two exons) in *NbWAKL5*, *NbWAKL6*, *NbWAKL11*, *NbWAKL17*, *NbWAKL20*, and *NbWAKL22*. Interestingly, most *NbWAK/WAKLs* in the same phylogenetic group had the same number of exons, such as *NbWAK1*, *NbWAK2*, *NbWAK4*, *NbWAK10*, *NbWAK11*, and *NbWAKL8* in Group I and *NbWAKL5*, *NbWAKL11*, *NbWAKL17*, *NbWAKL20*, and *NbWAKL22* in Group V (Fig. 3). These results suggest a similar diversity of expansion and evolution among members of the same phylogenetic group of *NbWAK/WAKLs* in *N. benthamiana*.

### Promoter analysis of *NbWAK/WAKL* genes

**Fig. 4:**
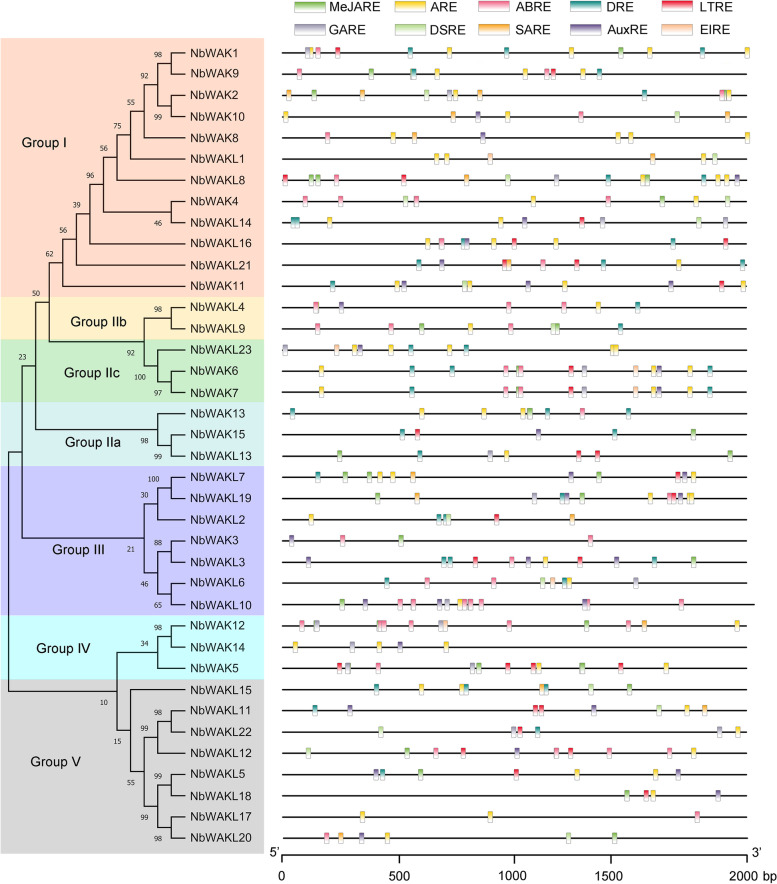
Schematic representation of molecular phylogenetic relationships and 2000 bp promoters of *WAK/WAKLs* in *Nicotiana benthamiana*. The phylogenetic tree was constructed using the Maximum-likelihood method with 1000 bootstrap replicates for each branch through MEGA 11.0. The 2000 bp promoter sequences of *NbWAK/WAKLs* were analyzed using PlantCARE and visualized using TBtools (v.1.098661). Different colored boxes represent different *cis*-acting elements

To explore the potential function and regulatory model of these *NbWAK/WAKL* genes, the *cis*-acting elements in the 2000 bp promoter sequences of *NbWAK/WAKLs* were analyzed using the PlantCARE database [51]. As a result, several phytohormone and/or stress response-related *cis*-acting elements were identified: MeJA-responsive element (MeJARE), anaerobic response element (ARE), ABA-responsive element (ABRE), drought-responsive element (DRE), low-temperature-responsive element (LTRE), gibberellin-responsive element (GARE), defense- and stress-responsive element (DSRE), SA-responsive element (SARE), auxin-responsive element (AuxRE), and elicitor-responsive element (EIRE) (Fig. 4 and Additional file 2). Out of the 38 *NbWAK/WAKLs*, 36 had the ARE element, 25 possessed the DRE element, 24 contained the ABRE element, 23 harbored the MeJARE and GARE elements, 20 contained the LTRE element, 15 possessed the SARE and AuxRE elements, 13 had the DSRE element, and 6 harbored the EIRE element (Fig. 4 and Additional file 2). These results indicate a possible involvement of these genes in various phytohormone and/or stress responses.

### Tissue-specific expression patterns of *NbWAK/WAKL* genes

**Fig. 5:**
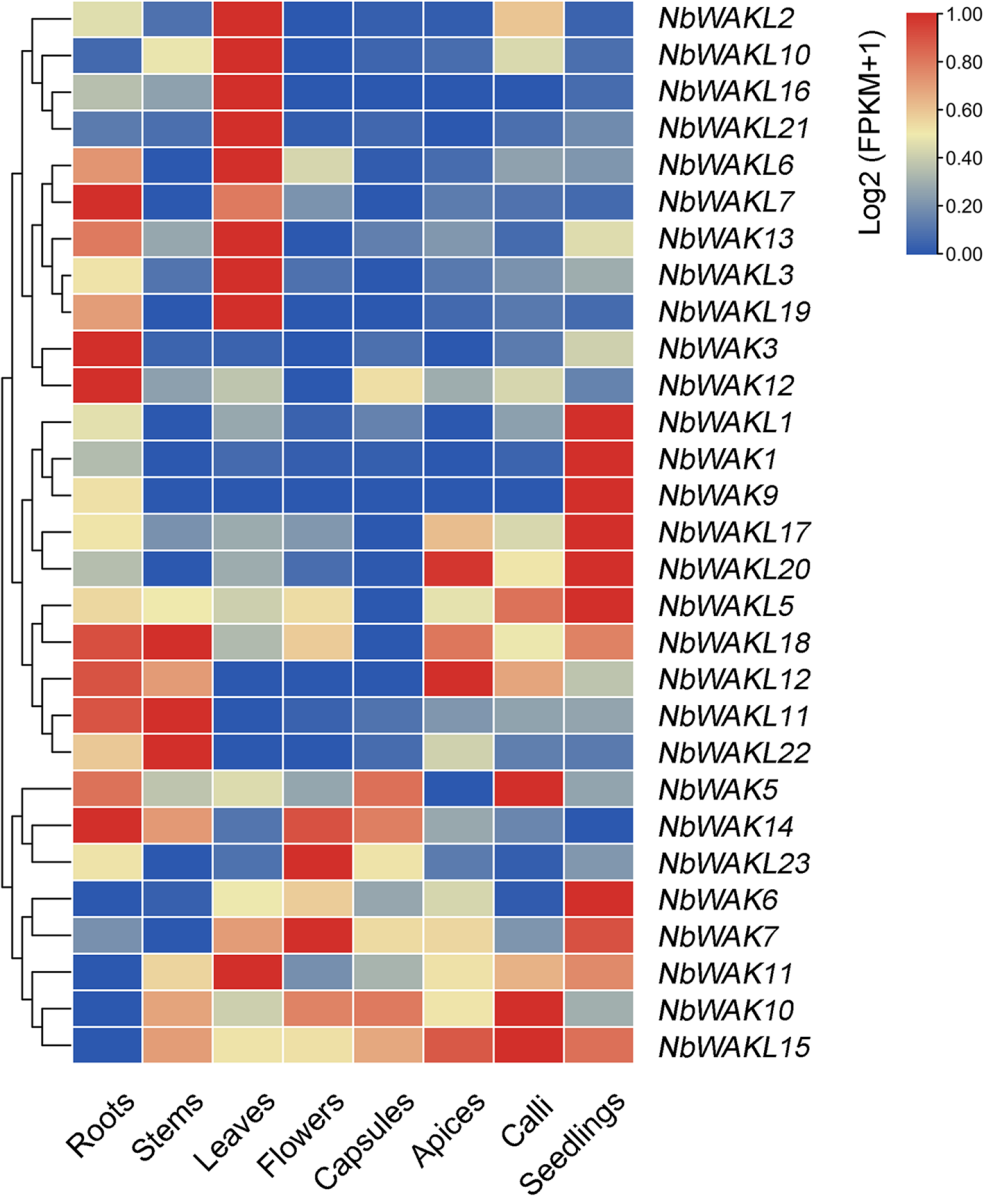
Expression profiles of *NbWAK/WAKLs* in different tissues of *Nicotiana benthamiana*. Transcriptomic data used for tissue expression were obtained from the NCBI sequence read archive (SRA) database (https://www.ncbi.nlm.nih.gov/sra/) under the accession number PRJNA188486 [52], and the expression level of each gene is colored based on their Log2 (FPKM+ 1) values calculated from eight tissues: roots, stems, leaves, flowers, capsules, apices, calli, and seedlings. The heatmap was generated using TBtools (v.1.098661)

To further investigate the expression patterns of *NbWAK/WAKLs* in *N. benthamiana*, we analyzed their expression profiles in different tissues (roots, stems, leaves, flowers, capsules, apices, calli, and seedlings) using the public RNA-sequencing data [52]. Expression analysis indicated that 29 of the 38 *NbWAK/WAKLs* were expressed in at least one tissue (Fig. 5 and Additional file 3). Seven genes (*NbWAK5*–*7*, *NbWAK10*, and *NbWAK12*–*14*) were detected in all examined tissues with fragments per kilobase of transcript per million mapped reads (FPKM) values ≥1.0 (Fig. 5 and Additional file 3). There were 11 genes (*NbWAK3*, *NbWAK5*–*7*, *NbWAK12*–*14*, *NbWAKL2*, *NbWAKL3*, *NbWAKL6*, and *NbWAKL11*) and 7 genes (*NbWAK6*, *NbWAK10*, *NbWAK11*, *NbWAK13*, *NbWAKL11*, *NbWAK14*, and *NbWAKL22*) that were highly expressed in the roots and stems of *N. benthamiana*, respectively (FPKM ≥3.0) (Fig. 5 and Additional file 3). In addition, 12 genes (*NbWAK6*, *NbWAK7*, *NbWAK10*–*13*, *NbWAKL2*, *NbWAKL3*, *NbWAKL6*, *NbWAKL10*, *NbWAKL19*, and *NbWAKL21*) were highly expressed in the leaves of *N. benthamiana* (FPKM ≥3.0), with the two highest expression levels noted for *NbWAK13* (FPKM ≥52.0) and *NbWAKL6* (FPKM ≥42.0) (Fig. 5 and Additional file 3). In the flower tissue of *N. benthamiana*, seven genes (*NbWAK6*, *NbWAK7*, *NbWAK10*, *NbWAK13*, *NbWAK14*, *NbWAKL6*, and *NbWAKL23*) showed high expression levels (FPKM ≥3.0), with the two highest expression levels noted for *NbWAK14* (FPKM ≥10.0) and *NbWAK7* (FPKM ≥7.0) (Fig. 5 and Additional file 3). These data suggest that each *NbWAK/WAKL* gene has a tissue-specific expression pattern, and such expression patterns may be related to their functions in regulating plant growth and development and stress responses.

### Expression profiles of *NbWAK/WAKL* genes following TYLCV infection

**Fig. 6:**
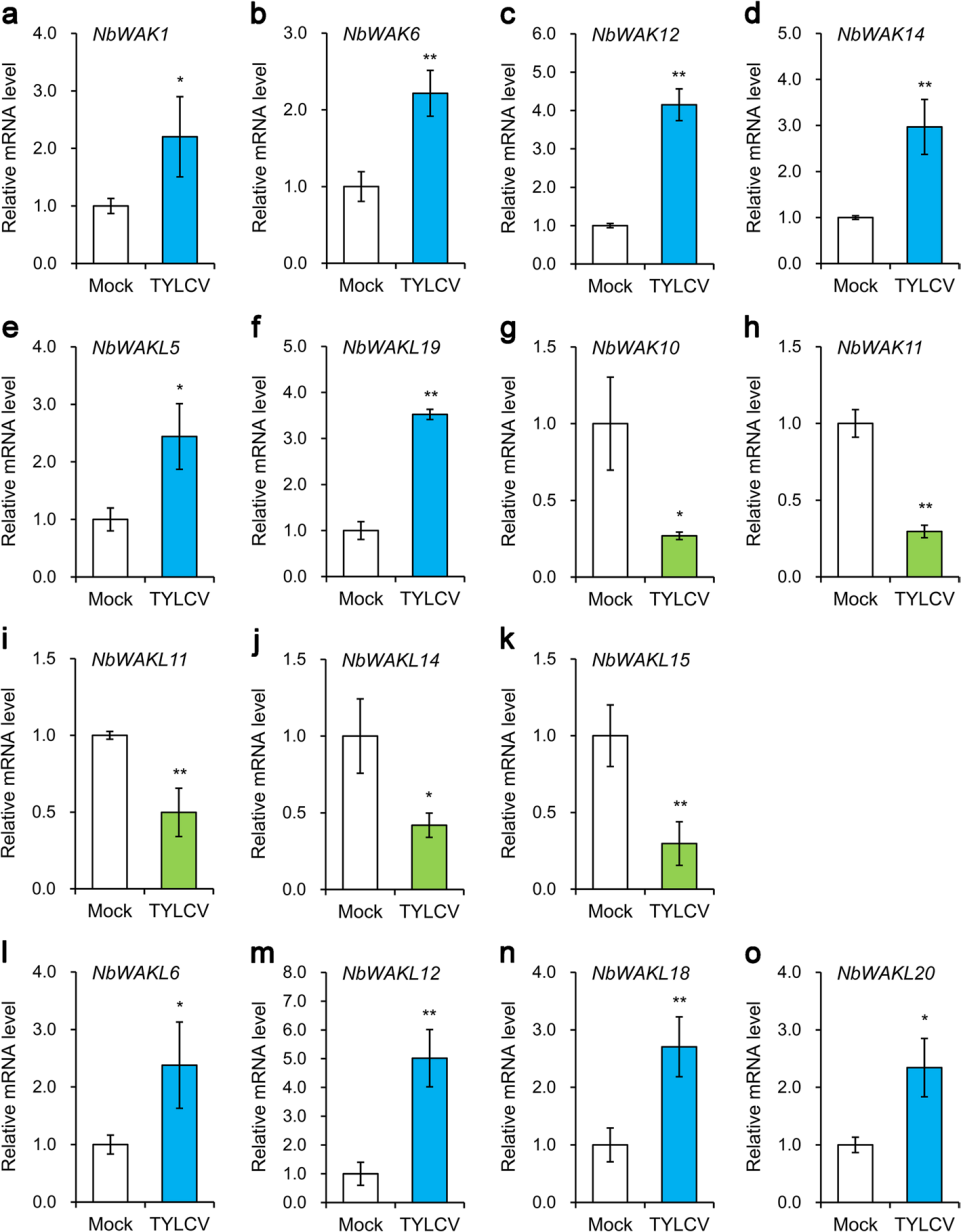
Expression levels of *NbWAKWAKLs* in leaves of *Nicotiana benthamiana* following tomato yellow leaf curl virus (TYLCV) infection. **a**–**k** Relative expression of *NbWAK1, NbWAK6, NbWAK12, NbWAK14, NbWAKL5, NbWAKL19, NbWAK10, NbWAK11, NbWAKL11, NbWAKL14,* and *NbWAKL15* in the locally infected leaves of N. benthamiana upon TYLCV infection. **l**–**o** Relative expression of *NbWAKL6, NbWAKL12, NbWAKL18,* and *NbWAKL20* in the systemically infected leaves of N. benthamiana upon TYLCV infection. Data represent relative mRNA levels against the leaves infected with Agrobacterium tumefaciens containing an empty vector (Mock), values of which are set to 1.0 units. The data are given as means ± standard deviation of three biological replicates. Statistically significant differences are marked with asterisks: * *P* < 0.05 or ** *P* < 0.01; Student’s *t*-test

To test whether *NbWAK/WAKLs* are involved in response to TYLCV infection, we examined the gene expression profiles of *NbWAK/WAKLs* in the leaves of *N. benthamiana* following TYLCV infection using expression data obtained from a previous study [53]. The results revealed that the expression of *NbWAK1*, *NbWAK6*, *NbWAK12*, *NbWAK14*, *NbWAKL5*, and *NbWAKL19* was upregulated significantly in the locally infected leaves of *N. benthamiana* following TYLCV infection (Fig. 6a–f). Furthermore, the expression of *NbWAK10*, *NbWAK11*, *NbWAKL11*, *NbWAKL14*, and *NbWAKL15* was downregulated dramatically in the locally infected leaves of *N. benthamiana* upon TYLCV infection (Fig. 6g–k). In contrast, the expression of *NbWAKL6*, *NbWAKL12*, *NbWAKL18*, and *NbWAKL20* was upregulated considerably in systemically infected leaves of *N. benthamiana* following TYLCV infection (Fig. 6l–o), and none of the *NbWAK/WAKLs* showed decreased expression levels in the systemic leaves of *N. benthamiana* in response to TYLCV. Interestingly, each of the upregulated *NbWAK/WAKLs* was increased more than two-fold in both the locally and systemically infected leaves of *N. benthamiana* following TYLCV infection (Fig. 6), indicating a critical role for these *NbWAK/WAKLs* in the response to TYLCV infection.

### Disruption of the expression of *NbWAK/WAKL* genes increases host susceptibility to TYLCV

**Fig. 7:**
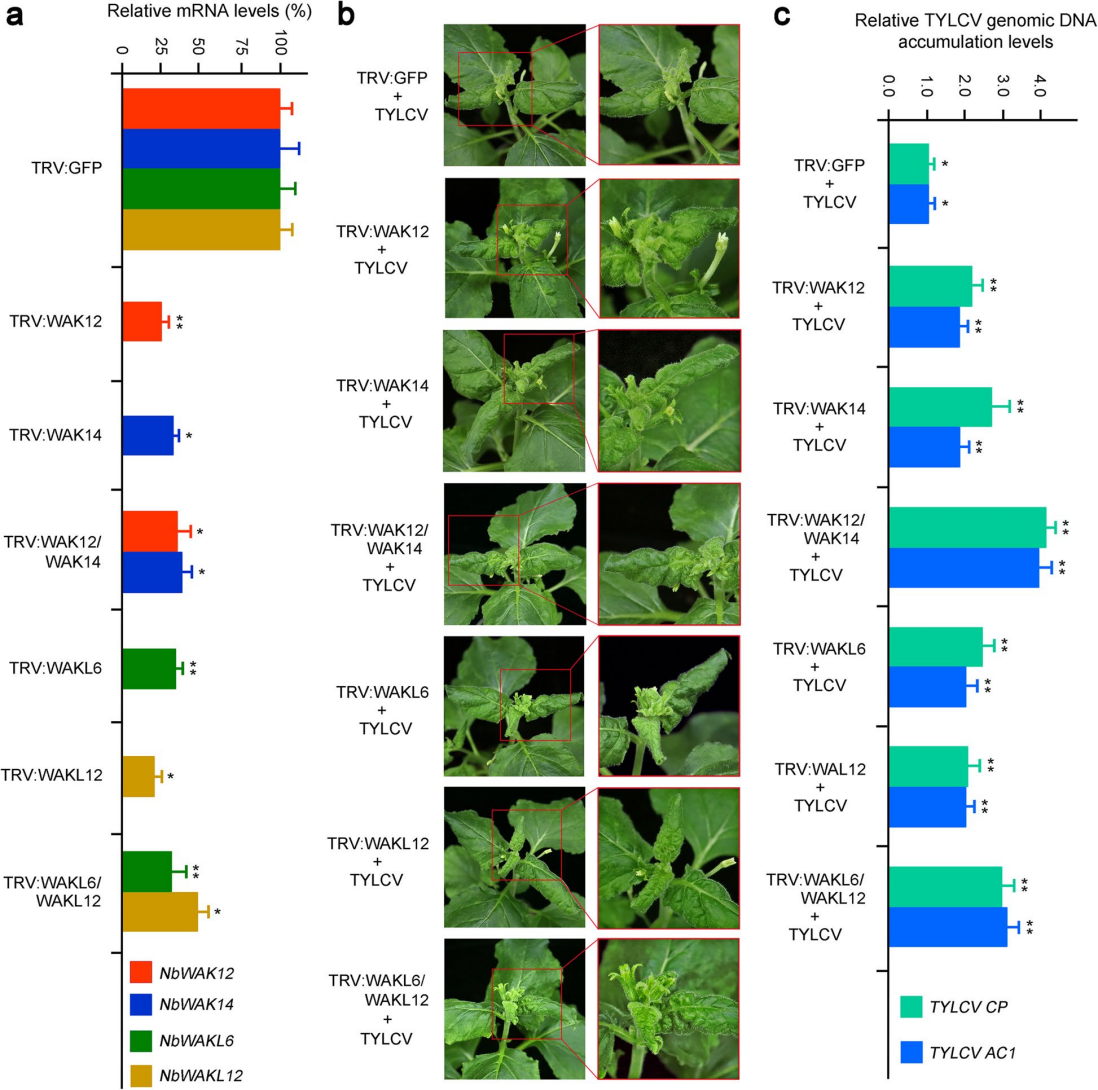
Silencing of the *NbWAK/WAKLs* in *Nicotiana benthamiana* makes it susceptible to infection with tomato yellow leaf curl virus (TYLCV). **a** Silencing efficiency was assessed by quantitative PCR (qPCR) at ten days post-inoculation (dpi). The values represent relative mRNA levels against those of the control groups (*N. benthamiana* seedlings agroinfiltrated with TRV:GFP), values of which are set to 100%. **b** Disease symptoms caused by TYLCV in the *NbWAK/WAKLs* silenced *N. benthamiana* seedlings at 21 dpi. TRV:GFP-agroinfiltrated *N. benthamiana* seedlings infected with TYLCV were used as the control. **c** Relative accumulation of TYLCV genomic DNA in the *NbWAK/WAKLs* silenced *N. benthamiana* seedlings. Viral accumulation was measured by qPCR at 21 dpi, as shown in Fig. 7a. The values represent relative viral DNA accumulation levels against those of the control groups (TRV:GFP-agroinfiltrated *N. benthamiana* seedlings infected with TYLCV), values of which are set to 1.0 units. For **a** and **c**, the data are given as means ± standard deviation of three biological replicates. Significant differences in expression are marked with asterisks: * *P* < 0.05 or ** *P* < 0.01; Student’s *t*-test

To further investigate the potential function of *NbWAK/WAKLs* in response to TYLCV infection, we examined their precise role in responding to TYLCV during viral infection using the VIGS technology [54]. Based on the tissue-specific expression patterns and gene expression profiles of *NbWAK/NbWAKLs* following TYLCV infection (Figs. 5 and 6), four genes, which included two *NbWAK* genes (*NbWAK12* and *NbWAK14*) and two *NbWAKL* genes (*NbWAKL6* and *NbWAKL12*), were selected and silenced individually or in combination with each other by TRV-based VIGS system. Compared with the vector control (*N. benthamiana* seedlings agroinfiltrated with TRV:GFP), the mRNA levels of *NbWAK12*, *NbWAK14*, *NbWAKL6*, and *NbWAKL12* in seedlings agroinfiltrated with the silencing vectors were decreased by approximately 40–80% at ten days post-infiltration (dpi) (Fig. 7a), suggesting that VIGS successfully silenced the target genes. Subsequently, the control and silenced seedlings were agroinfiltrated with the infectious clone of TYLCV and monitored for symptom development over time. At 21 dpi, compared with the vector control, severe leaf curling and crinkling symptoms caused by TYLCV were observed in systemic leaves of seedlings in which *NbWAK/NbWAKLs* were silenced by VIGS, especially in seedlings in which the two components of *NbWAK/WAKLs* were silenced (Fig. 7b). These results indicated that *N. benthamiana* seedlings were more susceptible to TYLCV when the *NbWAK/WAKL* genes were suppressed. For further confirmation, we determined the TYLCV genomic DNA accumulation by calculating the expression levels of *TYLCV CP* and *AC1* using quantitative PCR (qPCR), as established previously [55, 56]. Notably, *N. benthamiana* seedlings with individual or double silencing of *NbWAK/NbWAKLs* accumulated more viral genomic DNA than the control plants (Fig. 7c). Altogether, these results suggest that silencing of the components of *NbWAK/WAKLs* impairs the resistance of *N. benthamiana* to TYLCV and increases the accumulation of TYLCV genomic DNA in the host plants.

## Discussion

The *WAK/WAKL* gene family is a subset of RLKs that has critical roles in plant growth and development and resistance to abiotic and biotic stresses [30, 38, 39]. Here, we identified and characterized the *WAK/WAKL* gene family in *N. benthamiana*. This *WAK/WAKL* gene family consists of 38 *NbWAK/WAKL* members, which is more than that observed in tomato [48], *Arabidopsis* [28], cotton [33], potato [57], and common walnut [58] and less than that found in rice [59], *Populus* [60], apple [61], Chinese cabbage [62], barley [63], and rose [64]. Based on the amino acid sequences and phylogenetic relationships with the WAK/WAKL proteins in *Arabidopsis* and tomato, the 38 *NbWAK/WAKLs* were divided into five groups (Fig. 1). This result is consistent with the findings of previous studies on *WAK/WAKLs* from other plant species [31, 58, 63, 64]. Interestingly, Groups I and II were composed of *WAK/WAKL* genes from different plant species, including *N. benthamiana*, *Arabidopsis*, and tomato, whereas Groups III, IV, and V consisted of *WAK/WAKL* genes from *N. benthamiana* and tomato, suggesting that these *WAK/WAKL* genes of *N. benthamiana* and tomato may have evolved independently after the formation of the *WAK/WAKL* genes of *Arabidopsis*. This finding corroborates previous studies in which a specific set of *WAK/WAKL* genes in cotton had evolved independently [31, 65]. In addition, this classification of *NbWAK/WAKLs* was also supported by the conserved motif and gene structure analyses, showing that each phylogenetic group shares similar motifs and exon–intron structures (Figs. 2 and 3).

Previous studies have demonstrated that exon–intron structures frequently affect the evolution of a gene family [31, 66, 67]. In this study, gene structure analysis showed that different exon–intron structure patterns exist in different phylogenetic groups of *NbWAK/WAKLs*. The average number of exons of *NbWAK/WAKLs* was five in Group I, four in Groups II and IV, three in Group III, and two in Group V, respectively (Fig. 3). This finding aligns with the exon–intron structure of *WAK/WAKL* genes from *Populus* [60], tomato [48], and cotton [33], which is the result of the continuous evolution of the *WAK/WAKL* gene family. Furthermore, to obtain additional information about the regulation of *NbWAK/WAKLs*, we explored the *cis*-acting elements in their 2000 bp promoter sequences. Ten types of phytohormone and/or stress response-related *cis*-acting elements were identified, namely, MeJARE, ARE, ABRE, DRE, LTRE, GARE, DSRE, SARE, AuxRE, and EIRE (Fig. 4). This wide range of *cis*-acting elements is in line with the observations in previous studies on the phytohormone- and/or stress-responsive functions of *WAK/WAKL* genes in plants [33, 47, 48]. MeJARE, ABRE, GARE, SARE, and AuxRE were identified to involve in plant responses to MeJA, ABA, GA, SA, and auxin, respectively. This result suggests that *NbWAK/WAKLs* can be regulated by multiple plant hormones and thereby play a regulatory role in stress responses [33, 58]. Moreover, we also identified some *cis*-acting elements associated with stress responses in the promoter regions of *NbWAK/WAKLs*, such as ARE, DRE, LTRE, DSRE, and EIRE, suggesting that *NbWAK/WAKLs* may play a critical role in plant responses to different stresses [48, 68].

In addition, gene expression patterns frequently provide crucial information regarding gene functions [69]. Therefore, expression levels of *NbWAK/WAKLs* in the roots, stems, leaves, flowers, capsules, apices, calli, and seedlings were determined using public RNA-sequencing data [52]. The results indicated that most of the *NbWAK/WAKLs* were expressed in at least one tissue (Fig. 5), suggesting they may have essential roles in plant growth and development in *N. benthamiana*. These results corroborate the findings of previous studies in which *WAK/WAKLs* were found to have housekeeping functions in plant growth and development [32, 33, 48, 58]. In addition, relatively high expression levels of some genes were detected in the specific tissues, such as *NbWAK12*, *NbWAK13*, *NbWAK14*, and *NbWAKL6* in the roots, *NbWAK13* in the stems, *NbWAK11*, *NbWAK13*, *NbWAKL2*, *NbWAKL3*, and *NbWAKL6* in the leaves, and *NbWAK14* in flowers (Fig. 5 and Additional file 3), suggesting their potential functional implications in these tissues.

TYLCV is an important virus that can cause a severe yellow leaf curl disease in tomatoes and tobacco worldwide [10, 11]. To investigate whether *NbWAK/WAKLs* are involved in the response of *N. benthamiana* to TYLCV, we analyzed the expression profiles of *NbWAK/WAKLs* in locally and systemically infected leaves of *N. benthamiana* following TYLCV infection. Here, there were six and four *NbWAK/WAKLs* in the locally and systemically infected leaves of *N. benthamiana*, respectively, which were upregulated upon TYLCV infection (Fig. 6). These genes were unevenly distributed across each phylogenetic group, in which eight *NbWAK/WAKLs* (*NbWAK12*, *NbWAK14*, *NbWAKL5*, *NbWAKL6*, *NbWAKL12*, and *NbWAKL18*–*20*) belonged to the Groups III, IV, and V (Fig. 1) that have evolved independently during long-term evolution. We speculated that these independently evolved *NbWAKLs* play a crucial role in defending against viral infections. Consequently, four genes, including two *NbWAK* genes (*NbWAK12* and *NbWAK14*) and two *NbWAKL* genes (*NbWAKL6* and *NbWAKL12*), were silenced and then functionally determined through their response to TYLCV infection. Furthermore, the VIGS and qPCR results revealed that the accumulation of TYLCV genomic DNA was significantly increased when *NbWAK12*, *NbWAK14*, *NbWAKL6*, and *NbWAKL12* were silenced (Fig. 7), suggesting that these *NbWAK/WAKLs* play a critical role in the response to TYLCV infection. This result corroborates the results from previous studies in which a specific set of *WAK/WAKL* genes was shown to be strongly induced by pathogens in rice [40], cotton [46], and rose [64]. Although the precise functions of *NbWAK/WAKLs* in response to TYLCV infection are yet to be elucidated, our findings may help to clarify the expansion of the *NbWAK/WAKL* gene family and to characterize their function in resistance against TYLCV.

## Conclusions

Here, we described the first integrated investigation of the *WAK/WAKL* gene family in *N. benthamiana* through gene identification and the analysis of conserved motifs, gene structures, promoters, and tissue and TYLCV response expression profiles. A total of 38 *NbWAK/WAKLs* were identified on a genome-wide scale, which can provide essential information to functionally characterize the *WAK/WAKL* gene family in *N. benthamiana*. In addition, our VIGS and qPCR data demonstrated that TYLCV genomic DNA accumulation significantly increased when the four *NbWAK/WAKLs* (*NbWAK12*, *NbWAK14*, *NbWAKL6*, and *NbWAKL12*) were silenced in *N. benthamiana*. These findings are helpful to explore further the *WAK/WAKL* gene-mediated molecular processes implicated in response to TYLCV infection and to provide a basis for systematically investigating the functional mechanisms of the *WAK/WAKL* gene family in *N. benthamiana*.

## Methods

### Identification of *WAK/WAKL* genes in *N. benthamiana*

Genome sequences of *N. benthamiana* were obtained from the Sol Genomics Network (https://solgenomics.net/organism/Nicotiana_benthamiana/genome/) [70]. Protein sequences of WAK and WAKL proteins in *Arabidopsis* and tomato were downloaded from the Arabidopsis Information Resource version 11 (Araport11) (https://www.arabidopsis.org/) and International Tomato Genome Sequencing Consortium version 4.0 (ITAG 4.0) (https://solgenomics.net/organism/Solanum_lycopersicum/genome/), respectively [28, 48]. The known WAK and WAKL protein sequences (Additional file 1) were utilized to construct the HMM profile that was used to query the *N. benthamiana* protein dataset using HMMER software (v.3.2.1) [71]. The identified WAK and WAKL proteins from *N. benthamiana* were further confirmed by the presence of GUB_WAK_bind (PF13947), EGF (PF00008), and Pkinase (PF00069) domains using the Pfam database (http://pfam.xfam.org/) [72]. Candidate proteins that contained intact GUB_WAK_bind, EGF, and Pkinase domains were identified as NbWAKs; those that included two of these three domains were identified as NbWAKLs; those that had only one of these three domains were removed. The candidates, *NbWAK*/*WAKL* genes, identified from *N. benthamiana*, were named according to their corresponding physical map locations. The pI and MW of NbWAK/WAKL proteins were analyzed using the Compute pI/Mw tool (https://web.expasy.org/compute_pi/).

### Phylogenetic tree construction and protein motif analysis

The 38 NbWAK/WAKL protein sequences were used to establish evolutionary relationships with the known WAK/WAKL proteins from *Arabidopsis* and tomato (Additional file 1). Sequence alignment of these WAK/WAKL protein sequences was carried out using the MUSCLE program in MEGA 11.0 [73], and the phylogenetic tree was built with a ML method [74] based on the alignment through MEGA 11.0. Conserved motifs in NbWAK/WAKL protein sequences were analyzed using the MEME program (http://meme-suite.org/tools/meme/) [49] with the following parameters: the maximum motif number was set to 15, and the optimal motif width was set between 6 and 60.

### Gene structure and promoter analyses

Gene structure and promoter data for *NbWAK/WAKLs* were obtained from the Sol Genomics Network (https://solgenomics.net/organism/Nicotiana_benthamiana/genome/) [70]. Gene structure analysis was performed using the GSDS 2.0 (http://gsds.gao-lab.org/) [50]. Promoter analysis was performed by searching 2000 bp sequences upstream from the start codon of *NbWAK/WAKLs* against the PlantCARE database (http://bioinformatics.psb.ugent.be/webtools /plantcare/html/) [51] to identify putative *cis*-elements as described by Zhao et al. [75]. The MeJARE, ARE, ABRE, DRE, LTRE, GARE, DSRE, SARE, AuxRE, and EIRE related to phytohormone and/or stress responses were further analyzed. The results of the promoter analysis were visualized using TBtools (v.1.098661) [76].

### Tissue-specific and differential expression analyses

To determine the expression profiles of *NbWAK/WAKLs* in different tissues of *N. benthamiana*, a comparative analysis of published RNA-sequencing data, SRR696961, SRR696992, SRR696940, SRR696938, SRR696884, SRR685298, SRR697013, and SRR696988 [52], was carried out. Sequence assembly was conducted using the HISAT2 (v.2.1.0) [77], and the FPKM values were calculated using the StringTie2 (v.2.1.5) [78]. Different tissues in *N. benthamiana*, including the root, stem, leaf, flower, capsule, apex, calli, and seedling, were selected [52]. The results of the expression levels of *NbWAK/WAKLs* were visualized using TBtools (v.1.098661) [76]. For differential expression analysis of *NbWAK/WAKLs* in response to TYLCV, the gene expression data were obtained from a previous study [53] and re-analyzed using Microsoft Excel (v.2019, Microsoft Corp., USA), as described previously [69].

### Plant materials and growth conditions

Wild-type *N. benthamiana* plants were utilized in this study, and they were grown in a greenhouse belonging to Dr. Zhanqi Wang (Huzhou University) at 25 ± 1 °C with a 16 h/8 h (light/dark) photoperiod as described by Zhong et al. [1]. *N. benthamiana* seedlings at the 4- to 6-leaf stage were used for the experiments.

### VIGS and silencing efficiency assay

To silence the expression of *NbWAK12*, *NbWAK14*, *NbWAKL12*, and *NbWAKL6* genes in *N. benthamiana*, a tobacco rattle virus (TRV)-based VIGS system [54] was used. Approximately 300 bp fragments of *WAK/WAKL* genes were cloned individually into the KpnI-BamHI sites of the pTRV2 vector to generate VIGS constructs as described previously [6, 79]. The resulting VIGS plasmids were transformed into *Agrobacterium tumefaciens* strain EHA105 by electroporation, and *Agrobacterium*-mediated infiltration of *N. benthamiana* was carried out as described previously [6]. *N. benthamiana* seedlings agroinfiltrated with pTRV2:GFP and pTRV1 were used as the control. At ten dpi, the silencing efficiency of the VIGS was evaluated by qPCR analysis as described previously [1, 80]. The primers used for the VIGS constructs and qPCR are listed in Additional file 4.

### TYLCV inoculation and viral DNA accumulation

The infectious clone of TYLCV was gifted by Prof. Yan Xie (Zhejiang University, China). Viral inoculation with TYLCV on *N. benthamiana* was performed as described previously [53]. At 21 dpi, the inoculated *N. benthamiana* seedlings were photographed, and the systemically infected leaves were sampled. The accumulation of *TYLCV CP* and *AC1* was measured using the qPCR, and the *N. benthamiana 25S nuclear rRNA* gene (*Nb25SrRNA*) was used as endogenous control [1, 80]. The relative viral DNA accumulation levels were determined using a comparative threshold cycle (C_T_) method, and the data were from three independent biological replicates. The primers used for qPCR are listed in Additional file 4.

### Statistical analysis

All experiments were performed in three independent replicates, and the data were given as means ± standard deviation (SD). The statistical significance of differences was calculated using Student’s *t*-test, and a *P* value < 0.05 was considered statistically significant.

## Supplementary Information

**Additional file 1: Table S1.** Protein sequences of WAK/WAKL of *Nicotiana benthamiana*, *Arabidopsis*, and tomato.

**Additional file 2: Table S2.** *Cis*-acting elements in the promoter regions of *NbWAK/WAKL* genes.

**Additional file 3: Table S3.** Tissue expression profiles of *NbWAK/WAKL* genes.

**Additional file 4: Table S4.** List of primers used in this study.

## Data Availability

The datasets supporting the conclusions of this article are included within the article and its additional files.

## Abbreviations

ABA: Abscisic acid
ABRE: ABA-responsive element
ARE: Anaerobic response element
AuxRE: Auxin-responsive element
dpi: Days post-infiltration
DRE: Drought-responsive element
DSRE: Defense- and stress-responsive element
EGF: Epidermal growth factor
EIRE: Elicitor-responsive element
FPKM: Fragments per kilobase of transcript per million mapped reads
GARE: Gibberellin-responsive element
GSDS: Gene Structure Display Server
HMM: Hidden Markov model
JA: Jasmonic acid
LTRE: Low-temperature-responsive element
MeJARE: MeJA-responsive element
MEME: Multiple Em for Motif Elicitation
ML: Maximum-likelihood method
MW: Molecular weight
pI: Isoelectric point
PTGS: Post-transcriptional gene silencing
qPCR: Quantitative PCR
RLK: Receptor-like kinase
SA: Salicylic acid
SARE: SA-responsive element
TGS: Transcriptional gene silencing
TYLCV: Tomato yellow leaf curl virus
WAK: Wall-associated Kinase
WAKL: WAK-like Kinase
WAK/WAKL: WAK and WAKL

## References

[1] Zhong X, Wang ZQ, Xiao R, Cao L, Wang Y, Xie Y (2017). Mimic phosphorylation of a βC1 protein encoded by TYLCCNB impairs its functions as a viral suppressor of RNA silencing and a symptom determinant. J Virol.

[2] Li H, Zeng R, Chen Z, Liu X, Cao Z, Xie Q (2018). *S*-acylation of a geminivirus C4 protein is essential for regulating the CLAVATA pathway in symptom determination. J Exp Bot.

[3] Walker PJ, Siddell SG, Lefkowitz EJ, Mushegian AR, Adriaenssens EM, Alfenas-Zerbini P (2021). Changes to virus taxonomy and to the International Code of Virus Classification and Nomenclature ratified by the International Committee on Taxonomy of Viruses (2021). Arch Virol.

[4] Rojas MR, Macedo MA, Maliano MR, Soto-Aguilar M, Souza JO, Briddon RW (2018). World management of geminiviruses. Annu Rev Phytopathol.

[5] Osterbaan LJ, Fuchs M (2019). Dynamic interactions between plant viruses and their hosts for symptom development. J Plant Pathol.

[6] Zhong X, Wang ZQ, Xiao R, Wang Y, Xie Y, Zhou X (2017). TRAQ analysis of the tobacco leaf proteome reveals that RNA-directed DNA methylation (RdDM) has important roles in defense against geminivirus-betasatellite infection. J Proteome.

[7] García-Arenal F, Zerbini FM (2019). Life on the edge: geminiviruses at the interface between crops and wild plant hosts. Annu Rev Virol.

[8] Farooq T, Umar M, She X, Tang Y, He Z (2021). Molecular phylogenetics and evolutionary analysis of a highly recombinant begomovirus, cotton leaf curl Multan virus, and associated satellites. Virus Evol.

[9] Beam K, Ascencio-Ibáñez JT (2020). Geminivirus resistance: a minireview. Front Plant Sci.

[10] Prasad A, Sharma N, Hari-Gowthem G, Muthamilarasan M, Prasad M (2020). Tomato yellow leaf curl virus: impact, challenges, and management. Trends Plant Sci.

[11] Marchant WG, Gautam S, Dutta B, Srinivasan R (2022). Whitefly-mediated transmission and subsequent acquisition of highly similar and naturally occurring tomato yellow leaf curl virus variants. Phytopathology..

[12] Gong P, Tan H, Zhao S, Li H, Liu H, Ma Y (2021). Geminiviruses encode additional small proteins with specific subcellular localizations and virulence function. Nat Commun.

[13] Gong P, Zhao S, Liu H, Chang Z, Li F, Zhou X (2022). Tomato yellow leaf curl virus V3 protein traffics along microfilaments to plasmodesmata to promote virus cell-to-cell movement. Sci China Life Sci.

[14] Zhao S, Gong P, Ren Y, Liu H, Li H, Li F (2022). The novel C5 protein from tomato yellow leaf curl virus is a virulence factor and suppressor of gene silencing. Stress Biol.

[15] Glick E, Zrachya A, Levy Y, Mett A, Gidoni D, Belausov E (2008). Interaction with host SGS3 is required for suppression of RNA silencing by tomato yellow leaf curl virus V2 protein. Proc Natl Acad Sci U S A.

[16] Wang B, Yang X, Wang Y, Xie Y, Zhou X (2018). Tomato yellow leaf curl virus V2 interacts with host histone deacetylase 6 to suppress methylation-mediated transcriptional gene silencing in plants. J Virol.

[17] Wang L, Ding Y, He L, Zhang G, Zhu JK, Lozano-Duran R (2020). A virus-encoded protein suppresses methylation of the viral genome through its interaction with AGO4 in the Cajal body. Elife..

[18] Zhao W, Wu S, Barton E, Fan Y, Ji Y, Wang X (2020). Tomato yellow leaf curl virus V2 protein plays a critical role in the nuclear export of V1 protein and viral systemic infection. Front Microbiol.

[19] Rosas-Diaz T, Zhang D, Fan P, Wang L, Ding X, Jiang Y (2018). A virus-targeted plant receptor-like kinase promotes cell-to-cell spread of RNAi. Proc Natl Acad Sci U S A.

[20] Garnelo Gómez B, Zhang D, Rosas-Díaz T, Wei Y, Macho AP, Lozano-Durán R (2019). The C4 protein from tomato yellow leaf curl virus can broadly interact with plant receptor-like kinases. Viruses..

[21] Corrales-Gutierrez M, Medina-Puche L, Yu Y, Wang L, Ding X, Luna AP (2020). The C4 protein from the geminivirus tomato yellow leaf curl virus confers drought tolerance in *Arabidopsis* through an ABA-independent mechanism. Plant Biotechnol J.

[22] Padmanabhan C, Zheng Y, Shamimuzzaman M, Wilson JR, Gilliard A, Fei Z (2022). The tomato yellow leaf curl virus C4 protein alters the expression of plant developmental genes correlating to leaf upward cupping phenotype in tomato. PLoS One.

[23] Macho AP, Lozano-Duran R (2019). Molecular dialogues between viruses and receptor-like kinases in plants. Mol Plant Pathol.

[24] Zhang H, Zhu J, Gong Z, Zhu JK (2022). Abiotic stress responses in plants. Nat Rev Genet.

[25] Wolf S (2017). Plant cell wall signalling and receptor-like kinases. Biochem J.

[26] Dievart A, Gottin C, Périn C, Ranwez V, Chantret N (2020). Origin and diversity of plant receptor-like kinases. Annu Rev Plant Biol.

[27] Kohorn BD (2001). WAKs; cell wall associated kinases. Curr Opin Cell Biol.

[28] Verica JA, He ZH (2002). The cell wall-associated kinase (WAK) and WAK-like kinase gene family. Plant Physiol.

[29] Kanneganti V, Gupta AK (2008). Wall associated kinases from plants - an overview. Physiol Mol Biol Plants.

[30] Kohorn BD (2016). Cell wall-associated kinases and pectin perception. J Exp Bot.

[31] Zhang Z, Ma W, Ren Z, Wang X, Zhao J, Pei X (2021). Characterization and expression analysis of wall-associated kinase (WAK) and WAK-like family in cotton. Int J Biol Macromol.

[32] Yang Z (2022). Plant growth: a matter of WAK seeing the wall and talking to BRI1. Curr Biol.

[33] Dou L, Li Z, Shen Q, Shi H, Li H, Wang W (2021). Genome-wide characterization of the WAK gene family and expression analysis under plant hormone treatment in cotton. BMC Genomics.

[34] Li L, Li K, Ali A, Guo Y (2021). AtWAKL10, a cell wall associated receptor-like kinase, negatively regulates leaf senescence in *Arabidopsis thaliana*. Int J Mol Sci.

[35] Sivaguru M, Ezaki B, He ZH, Tong H, Osawa H, Baluska F (2003). Aluminum-induced gene expression and protein localization of a cell wall-associated receptor kinase in *Arabidopsis*. Plant Physiol.

[36] Lou HQ, Fan W, Jin JF, Xu JM, Chen WW, Yang JL (2020). A NAC-type transcription factor confers aluminum resistance by regulating cell wall-associated receptor kinase 1 and cell wall pectin. Plant Cell Environ.

[37] Xia Y, Yin S, Zhang K, Shi X, Lian C, Zhang H (2018). OsWAK11, a rice wall-associated kinase, regulates Cu detoxification by alteration the immobilization of Cu in cell walls. Environ Exp Bot.

[38] Amsbury S (2020). Sensing attack: the role of wall-associated kinases in plant pathogen responses. Plant Physiol.

[39] Stephens C, Hammond-Kosack KE, Kanyuka K (2022). WAKsing plant immunity, waning diseases. J Exp Bot.

[40] Hu K, Cao J, Zhang J, Xia F, Ke Y, Zhang H (2017). Improvement of multiple agronomic traits by a disease resistance gene via cell wall reinforcement. Nat Plants.

[41] Saintenac C, Lee WS, Cambon F, Rudd JJ, King RC, Marande W (2018). Wheat receptor-kinase-like protein Stb6 controls gene-for-gene resistance to fungal pathogen *Zymoseptoria tritici*. Nat Genet.

[42] Gadaleta A, Colasuonno P, Giove SL, Blanco A, Giancaspro A (2019). Map-based cloning of *QFhb.mgb-2A* identifies a *WAK2* gene responsible for Fusarium Head Blight resistance in wheat. Sci Rep.

[43] Zuo W, Chao Q, Zhang N, Ye J, Tan G, Li B (2015). A maize wall-associated kinase confers quantitative resistance to head smut. Nat Genet.

[44] Yang P, Praz C, Li B, Singla J, Robert C, Kessel B (2019). Fungal resistance mediated by maize wall-associated kinase ZmWAK-RLK1 correlates with reduced benzoxazinoid content. New Phytol.

[45] Zhang N, Pombo MA, Rosli HG, Martin GB (2020). Tomato wall-associated kinase SlWak1 depends on Fls2/Fls3 to promote apoplastic immune responses to *Pseudomonas syringae*. Plant Physiol.

[46] Feng H, Li C, Zhou J, Yuan Y, Feng Z, Shi Y (2021). A cotton WAKL protein interacted with a DnaJ protein and was involved in defense against *Verticillium dahliae*. Int J Biol Macromol.

[47] Yang J, Xie M, Wang X, Wang G, Zhang Y, Li Z (2021). Identification of cell wall-associated kinases as important regulators involved in *Gossypium hirsutum* resistance to *Verticillium dahliae*. BMC Plant Biol.

[48] Sun Z, Song Y, Chen D, Zang Y, Zhang Q, Yi Y (2020). Genome-wide identification, classification, characterization, and expression analysis of the wall-associated kinase family during fruit development and under wound stress in tomato (*Solanum lycopersicum* L.). Genes..

[49] Bailey TL, Johnson J, Grant CE, Noble WS (2015). The MEME suite. Nucleic Acids Res.

[50] Hu B, Jin J, Guo AY, Zhang H, Luo J, Gao G (2015). GSDS 2.0: an upgraded gene feature visualization server. Bioinformatics..

[51] Lescot M, Déhais P, Thijs G, Marchal K, Moreau Y, Van de Peer Y (2002). PlantCARE, a database of plant *cis*-acting regulatory elements and a portal to tools for in silico analysis of promoter sequences. Nucleic Acids Res.

[52] Nakasugi K, Crowhurst RN, Bally J, Wood CC, Hellens RP, Waterhouse PM (2013). *De novo* transcriptome sequence assembly and analysis of RNA silencing genes of *Nicotiana benthamiana*. PLoS One.

[53] Wu M, Ding X, Fu X, Lozano-Duran R (2019). Transcriptional reprogramming caused by the geminivirus tomato yellow leaf curl virus in local or systemic infections in *Nicotiana benthamiana*. BMC Genomics.

[54] Liu Y, Schiff M, Dinesh-Kumar SP (2002). Virus-induced gene silencing in tomato. Plant J.

[55] Wang LL, Wang XR, Wei XM, Huang H, Wu JX, Chen XX (2016). The autophagy pathway participates in resistance to tomato yellow leaf curl virus infection in whiteflies. Autophagy..

[56] Yang Y, Liu T, Shen D, Wang J, Ling X, Hu Z (2019). Tomato yellow leaf curl virus intergenic siRNAs target a host long noncoding RNA to modulate disease symptoms. PLoS Pathog.

[57] Yu H, Zhang W, Kang Y, Fan Y, Yang X, Shi M, et al. Genome-wide identification and expression analysis of wall-associated kinase (WAK) gene family in potato (*Solanum tuberosum* L.). Plant Biotechnol Rep. 2022;16:317–31.

[58] Li M, Ma J, Liu H, Ou M, Ye H, Zhao P (2022). Identification and characterization of wall-associated kinase (WAK) and WAK-like (WAKL) gene family in *Juglans regia* and its wild related species *Juglans mandshurica*. Genes..

[59] Zhang S, Chen C, Li L, Meng L, Singh J, Jiang N (2005). Evolutionary expansion, gene structure, and expression of the rice wall-associated kinase gene family. Plant Physiol.

[60] Tocquard K, Lafon-Placette C, Auguin D, Muries B, Bronner G, Lopez D (2014). In silico study of wall-associated kinase family reveals large-scale genomic expansion potentially connected with functional diversification in *Populus*. Tree Genet Genomes.

[61] Zuo C, Liu Y, Guo Z, Mao J, Chu M, Chen B (2019). Genome-wide annotation and expression responses to biotic stresses of the WALL-ASSOCIATED KINASE - RECEPTOR-LIKE KINASE (WAK-RLK) gene family in apple (*Malus domestica*). Eur J Plant Pathol.

[62] Zhang B, Li P, Su T, Li P, Xin X, Wang W (2020). Comprehensive analysis of wall-associated kinase genes and their expression under abiotic and biotic stress in Chinese cabbage (*Brassica rapa* ssp. *pekinensis*). J Plant Growth Regul.

[63] Tripathi RK, Aguirre JA, Singh J (2021). Genome-wide analysis of wall associated kinase (WAK) gene family in barley. Genomics..

[64] Liu X, Wang Z, Tian Y, Zhang S, Li D, Dong W (2021). Characterization of wall-associated kinase/wall-associated kinase-like (WAK/WAKL) family in rose (*Rosa chinensis*) reveals the role of RcWAK4 in *Botrytis* resistance. BMC Plant Biol.

[65] Wang P, Zhou L, Jamieson P, Zhang L, Zhao Z, Babilonia K (2020). The cotton wall-associated kinase GhWAK7A mediates responses to fungal wilt pathogens by complexing with the chitin sensory receptors. Plant Cell.

[66] Zaynab M, Peng J, Sharif Y, Albaqami M, Al-Yahyai R, Fatima M (2022). Genome-wide identification and expression profiling of DUF221 gene family provides new insights into abiotic stress responses in potato. Front Plant Sci.

[67] Kuzmin E, VanderSluis B, Nguyen Ba AN, Wang W, Koch EN, Usaj M (2020). Exploring whole-genome duplicate gene retention with complex genetic interaction analysis. Science.

[68] Tan Z, Wen X, Wang Y (2020). *Betula platyphylla* BpHOX2 transcription factor binds to different cis-acting elements and confers osmotic tolerance. J Integr Plant Biol.

[69] Jin JF, Wang ZQ, He QY, Wang JY, Li PF, Xu JM (2020). Genome-wide identification and expression analysis of the NAC transcription factor family in tomato (*Solanum lycopersicum*) during aluminum stress. BMC Genomics.

[70] Bombarely A, Rosli HG, Vrebalov J, Moffett P, Mueller LA, Martin GB (2012). A draft genome sequence of *Nicotiana benthamiana* to enhance molecular plant-microbe biology research. Mol Plant-Microbe Interact.

[71] Mistry J, Finn RD, Eddy SR, Bateman A, Punta M (2013). Challenges in homology search: HMMER3 and convergent evolution of coiled-coil regions. Nucleic Acids Res.

[72] Mistry J, Chuguransky S, Williams L, Qureshi M, Salazar GA, Sonnhammer E (2021). Pfam: The protein families database in 2021. Nucleic Acids Res.

[73] Tamura K, Stecher G, Kumar S (2021). MEGA11: molecular evolutionary genetics analysis version 11. Mol Biol Evol.

[74] Leng ZX, Liu Y, Chen ZY, Guo J, Chen J, Zhou YB (2021). Genome-wide analysis of the DUF4228 family in soybean and functional identification of GmDUF4228-70 in response to drought and salt stresses. Front Plant Sci.

[75] Zhao W, Liu H, Zhang L, Hu Z, Liu J, Hua W (2019). Genome-wide identification and characterization of *FBA* gene family in polyploid crop *Brassica napus*. Int J Mol Sci.

[76] Chen C, Chen H, Zhang Y, Thomas HR, Frank MH, He Y (2020). TBtools: an integrative toolkit developed for interactive analyses of big biological data. Mol Plant.

[77] Kim D, Paggi JM, Park C, Bennett C, Salzberg SL (2019). Graph-based genome alignment and genotyping with HISAT2 and HISAT-genotype. Nat Biotechnol.

[78] Kovaka S, Zimin AV, Pertea GM, Razaghi R, Salzberg SL, Pertea M (2019). Transcriptome assembly from long-read RNA-seq alignments with StringTie2. Genome Biol.

[79] Luo C, Wang ZQ, Liu X, Zhao L, Zhou X, Xie Y (2019). Identification and analysis of potential genes regulated by an alphasatellite (TYLCCNA) that contribute to host resistance against tomato yellow leaf curl China virus and its betasatellite (TYLCCNV/TYLCCNB) infection in *Nicotiana benthamiana*. Viruses..

[80] Gui X, Liu C, Qi Y, Zhou X (2022). Geminiviruses employ host DNA glycosylases to subvert DNA methylation-mediated defense. Nat Commun.

